# Vitamin D receptor gene polymorphisms and multiple myeloma: a meta-analysis

**DOI:** 10.1007/s10238-024-01382-4

**Published:** 2024-06-04

**Authors:** Chunyi Lyu, Xuewei Yin, Zonghong Li, Teng Wang, Ruirong Xu

**Affiliations:** 1 Shandong University of Traditional Chinese Medicine, Jinan, People’s Republic of China; 2 Department of Hematology, Affiliated Hospital of Shandong University of Traditional Chinese Medicine, Jinan, People’s Republic of China; 3 Shandong Key Laboratory of Hematology of Integrated Traditional Chinese and Western Medicine of Health Commission, Institute of Hematology, Shandong University of Traditional Chinese Medicine, Jinan, People’s Republic of China

**Keywords:** Multiple myeloma, Gene polymorphism, Vitamin D receptor, Meta-analysis

## Abstract

Vitamin D acts through the vitamin D receptor (VDR), and vitamin D level decreases in multiple myeloma (MM) patients. Single nucleotide polymorphisms in VDR alter its functions to affect the vitamin D status. This raises the question of whether VDR gene polymorphisms are associated with MM risk, which has been investigated in case‒control studies, but the results have been inconsistent. This meta-analysis aimed to investigate the relationship between VDR gene polymorphisms and MM risk. The PubMed, Web of Science, Medline, Embase, Chinese National Knowledge Infrastructure (CNKI), Chinese Scientific Journal (VIP), Wanfang Databases (WANFANG) were searched from inception to June 1, 2023, without language restriction or publication preference. Pooled odds ratio (OR) and 95% confidence interval (CI) for each variable were calculated. Leave-one-out sensitivity analysis was performed to determine the source of heterogeneity. Publication bias was assessed using Begg’ and Egger’s tests, and the trim-and-fill method was used to compensate for publication bias. The correlation meta‐analysis was conducted using Comprehensive Meta‐Analysis 3.0 and STATA 12.0 software. All the included studies were based on Asian populations and involved four VDR gene polymorphisms, TaqI (rs731236), ApaI (rs7975232), BsmI (rs1544410) and FokI (rs2228570). The results showed that TaqI (C vs. T: OR = 1.487, 95% CI 1.052, 2.104,* P* = 0.025; CC + CT vs. TT: OR = 1.830, 95% CI 1.138, 2.944,* P* = 0.013), ApaI (T vs. G: OR = 1.292, 95% CI 1.101, 1.517, *P* = 0.002; TT vs. GG: OR = 1.600, 95% CI 1.106, 2.314, *P* = 0.013; TG vs. GG: OR 1.305, 95% CI 1.050, 1.622;* P* = 0.016; TT + TG vs. GG: OR = 1.353, 95% CI 1.103, 1.662, *P* = 0.004), BsmI (GG vs. AA: OR = 1.918, 95% CI 1.293, 2.844, *P* = 0.001; GA vs. AA: OR = 1.333, 95% CI 1.058, 1.679, *P* = 0.015; G vs. A: OR = 1.398, 95% CI 1.180, 1.657, *P* = 0.000; GG vs. AA + GA: OR = 1.686, 95% CI 1.174, 2.423, *P* = 0.005), and FokI (T vs. C: OR = 1.687, 95% CI 1.474, 1.931, *P* = 0.000; TT vs. CC: OR = 2.829, 95% CI 2.066, 3.872, *P* = 0.000; TC vs. CC: OR = 1.579, 95% CI 1.304, 1.913, *P* = 0.000, TT + TC vs. CC: OR = 1.771, 95% CI 1.477, 2.125,* P* = 0.000; TT vs. CC + TC: OR = 2.409, 95% CI 1.814, 3.200, *P* = 0.000) are associated with MM risk. VDR gene polymorphisms including ApaI, BsmI, TaqI, and FokI are associated with MM risk in Asian populations. Additional studies with large sample sizes and different ethnicities are needed.

**Supplementary Information:**

The online version contains supplementary material available at 10.1007/s10238-024-01382-4.

## Introduction

Multiple myeloma (MM) is a malignancy of clonal plasma cells that manifests clinically as bone pain, pathologic fractures, anemia, and renal insufficiency [1]. An increasing number of studies have reported that patients newly diagnosed with MM have low levels of vitamin D [2], and vitamin D deficiency is correlated with myeloma activity [3], the occurrence of peripheral neuropathy (PN) [4, 5], and poorer outcomes [6]. Evidence from laboratory has shown that analogs of vitamin D inhibit the growth of the myeloma cell line NCI-H929 via cell cycle arrest and apoptosis [7–11], and vitamin D supplements have potential for improving cancer prognosis and outcome in deficient individuals [12].

Vitamin D functions through its vitamin D receptor (VDR), and genetic variations of VDR could influence an individual’s vitamin D status [13, 14]. Polymorphisms caused by single nucleotides at the genome level are described as single nucleotide polymorphisms (SNPs), the most common form of variations among individuals. The most reported VDR gene polymorphisms are the BsmI (rs1544410), FokI (rs2228570), TaqI (rs731236) and ApaI (rs7975232) [15]. Studies have explored the association between these VDR gene polymorphisms and cancer development, and revealed that BsmI is associated with overall survival in patients with cancer, that ApaI is associated with progression-free survival in patients with cancer, and that FokI is associated with overall survival in patients with lung cancer [16]. In this study, we hypothesized that VDR gene polymorphisms could affect the binding of the VDR and have the potential to influence the risk of MM. MM is a multifactorial disease involving genetics [17], and factors such as the lifestyle and environmental exposures might change the actual effect of SNPs. Additionally, there is genetic heterogeneity in different ethnic populations [18]. These are uncertainties and conflicts that underlie the hypotheticals.

In MM, VDR gene polymorphisms have been investigated in several case‒control studies [19–24], and a meta-analysis indicated that the heterozygote and homozygote models of FokI and the homozygote model of ApaI are associated with an increased risk of MM [18]. However, conflicting conclusions have been reported in case‒control studies, and the associations of BsmI and TaqI with MM were not explored in previous meta-analysis. Further, significant heterogeneity was observed in the existing meta-analysis. In this study, we focused on the following clinical question: Is there an association between VDR gene polymorphisms and the risk of MM? This evidence will indicate the existence of VDR gene polymorphisms that may increase genetic susceptibility to MM in healthy individuals to promote earlier screening and diagnosis, and will provide evidence for developing therapies against VDR gene polymorphisms for MM patients. Therefore, a meta-analysis was conducted with the aim of examining and summarizing the evidence on the association between VDR gene polymorphisms and MM risk.

## Materials and methods

### Ethics

This study did not require patient recruitment or personal data collection, and there was no need for ethics committee approval.

### Protocol registration

This meta-analysis was conducted following the guidelines of the Preferred Reporting Items for Systematic Reviews and Meta-Analyses (PRISMA) 2020 checklist [25] and Cochrane Handbook criteria, and the protocol was registered with the International Platform of Registered Systematic Review and Meta-analysis Protocols (INPLASY) with registration number INPLASY202330076. The PRISMA 2020 checklist is provided in Supplementary file 1.

### Eligibility criteria and study selection

The research question was formulated using Population, Intervention, Comparison, Outcomes, and Study (PICOS) guidelines [26]. In the PICOS form, the studies were described as follows: P, patients diagnosed with MM; I, VDR gene polymorphisms; C, healthy control groups; O, effect of VDR gene polymorphisms associated with MM risk; S, case–control study. The inclusion criteria were as follows: (i) case‒control study in humans in which the association between VDR polymorphisms and MM risk was investigated; (ii) the case group included patients who meet the diagnostic criteria for MM, and the control group included healthy individuals; (iii) the number of individuals with each genotype in the case and control groups was sufficient to calculate odds ratio (OR) and 95% confidence interval (95% CI); and (iv) the genotype distributions of the control group followed the Hardy–Weinberg equilibrium (HWE). The exclusion criteria were as follows: (i) not a case–control study reporting the association between VDR gene polymorphisms and MM risk; (ii) studies containing data repeated from another published study; (iii) studies containing incomplete data or data that could not be analyzed; (iv) studies with missing or apparently incorrect data; and (v) reviews, case reports, or basic experimental studies.

### Literature search and extraction strategy

The PubMed, Web of Science, Medline, Embase, Chinese National Knowledge Infrastructure (CNKI), Chinese Scientific Journal Database (VIP), and Wanfang Database (WANFANG) were searched from their respective inception to June 1, 2023. The retrieval keywords were “multiple myeloma”, “vitamin D receptor”, and “polymorphism”. The retrieval strategy is provided in Supplementary file 2. Two reviewers independently screened and extracted the data. If there were any discrepancies, a third individual reviewed the articles. The data extracted included the following: first author, publication data, sample source, ethnicity, genotyping method, sample size, and genotype characterization for the cases and controls.

### Quality evaluation of the included studies

The quality of the studies included in this meta-analysis was evaluated using the Newcastle–Ottawa Scale (NOS) [27]. The full score was 9, and high quality was defined as a study with ≥ 7.

### Statistical analysis

The data for the meta-analysis and sensitivity analysis were analyzed using Comprehensive Meta-Analysis (CMA, version 3.0). The trim-and-fill method was used to test publication bias using STATA 12.0 (STATA Corporation, Texas, USA). According to whether the heterogeneity was low (I^2^ < 50), or high (I^2^ ≥ 50%), we used fixed- or random-effects models, respectively. The OR was used as a summary statistic for dichotomous variables. The 95% CI was calculated for all mean values. *P* values > 0.05 were considered not significant.

To explore the source of heterogeneity among the studies included in the review, a leave-one-out sensitivity analysis in which a meta-analysis was conducted on each subset of the studies obtained by leaving out exactly one study, was performed.Publication bias was tested using Begg’s and Egger’s tests. If *P* < 0.05, publication bias existed, and the trim-and-fill method was used to identify and correct for publication bias.

## Results

### Characteristics of the included studies

**Fig. 1:**
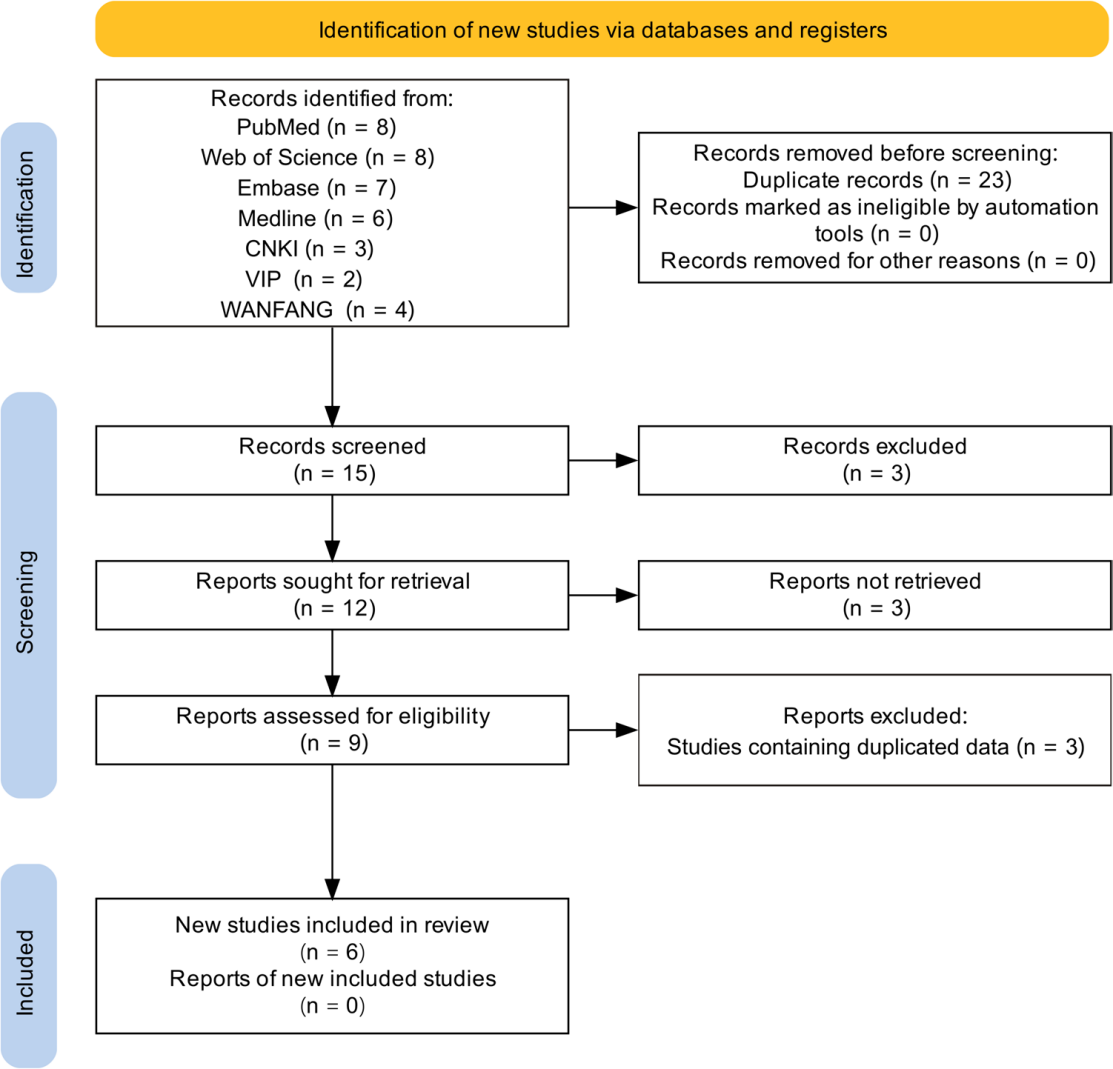
Flowchart of the literature-screening process

A total of 38 references were identified in the initial examination. After layer-by-layer screening, six case‒control studies were included in this analysis. Four case‒control studies with 342 cases and 336 controls were included for TaqI (rs731236), four case‒control studies with 689 cases and 1222 controls were included for ApaI (rs7975232), four case‒control studies with 689 cases and 1147 controls included for BsmI (rs1544410), and six case‒control studies with 877 cases and 1414 controls were included for FokI (rs2228570). The flow of the literature screening is detailed in Fig. 1. The included studies involved individuals of Asian ethnicity, four studies included individuals of Chinese Han ethnicity, one study included individuals of Indian ethnicity, and one study included individuals of Kashmiri ethnicity. PCR was the main genotyping method employed. The results of the quality assessment of the literature showed that the scores of the included studies ranged from ranged from 7 to 8 points, indicating that the studies were rated good or better. The basic characteristics of the included studies and the results of the quality evaluation are shown in Table 1. All genotype frequencies of the control group were consistent with the HWE. The genotype data are provided in Supplementary file 3.

**Table 1:** Basic characteristics of the included studies

References	Ethnicity	Genotyping method	Sample size (case/control)	Sample source	NOS score
FokI(rs2228570)					
Rui et al. [23]	Chinese Han population	PCR	40/84	Elbow vein blood	7
Ni [21]	Chinese Han population	PCR	114/60	Venous blood	8
Chen et al. [20]	Chinese Han population	PCR–RFLP	460/928	Blood	8
He et al. [19]	Chinese Han population	PCR	113/117	Venous blood	8
Kumar et al. [22]	Indian population	PCR–RFLP	75/75	Bone marrow Blood	7
Shafia et al. [24]	Kashmiri population	PCR	75/150	Peripheral blood	8
ApaI (rs7975232)					
Rui et al. [23]	Chinese Han population	PCR	40/84	Elbow vein blood	7
Ni [21]	Chinese Han population	PCR	114/60	Venous blood	8
Chen et al. [20]	Chinese Han population	PCR–RFLP	460/928	Blood	8
Shafia et al. [24]	Kashmiri population	PCR	75/150	Peripheral blood	8
BsmI (rs1544410)					
Rui et al. [23]	Chinese Han population	PCR	40/84	Elbow vein blood	7
Ni [21]	Chinese Han population	PCR	114/60	Venous blood	8
Chen et al. [20]	Chinese Han population	PCR–RFLP	460/928	Blood	8
Kumar et al. [22]	Indian population	PCR–RFLP	75/75	Bone marrow blood	7
TaqI (rs731236)					
Rui et al. [23]	Chinese Han population	PCR	40/84	Elbow vein blood	7
Ni [21]	Chinese Han population	PCR	114/60	Venous blood	8
He et al. [19]	Chinese Han population	PCR	113/117	Venous blood	8
Kumar et al. [22]	Indian population	PCR–RFLP	75/75	Bone marrow blood	7

*NOS* Newcastle–Ottawa Scale, *PCR* polymerase chain reaction, *PCR–RFLP* polymerase chain reaction-restriction fragment length polymorphism

### *Meta*-analysis results

#### Association between TaqI (rs731236) and MM risk

**Fig. 2:**
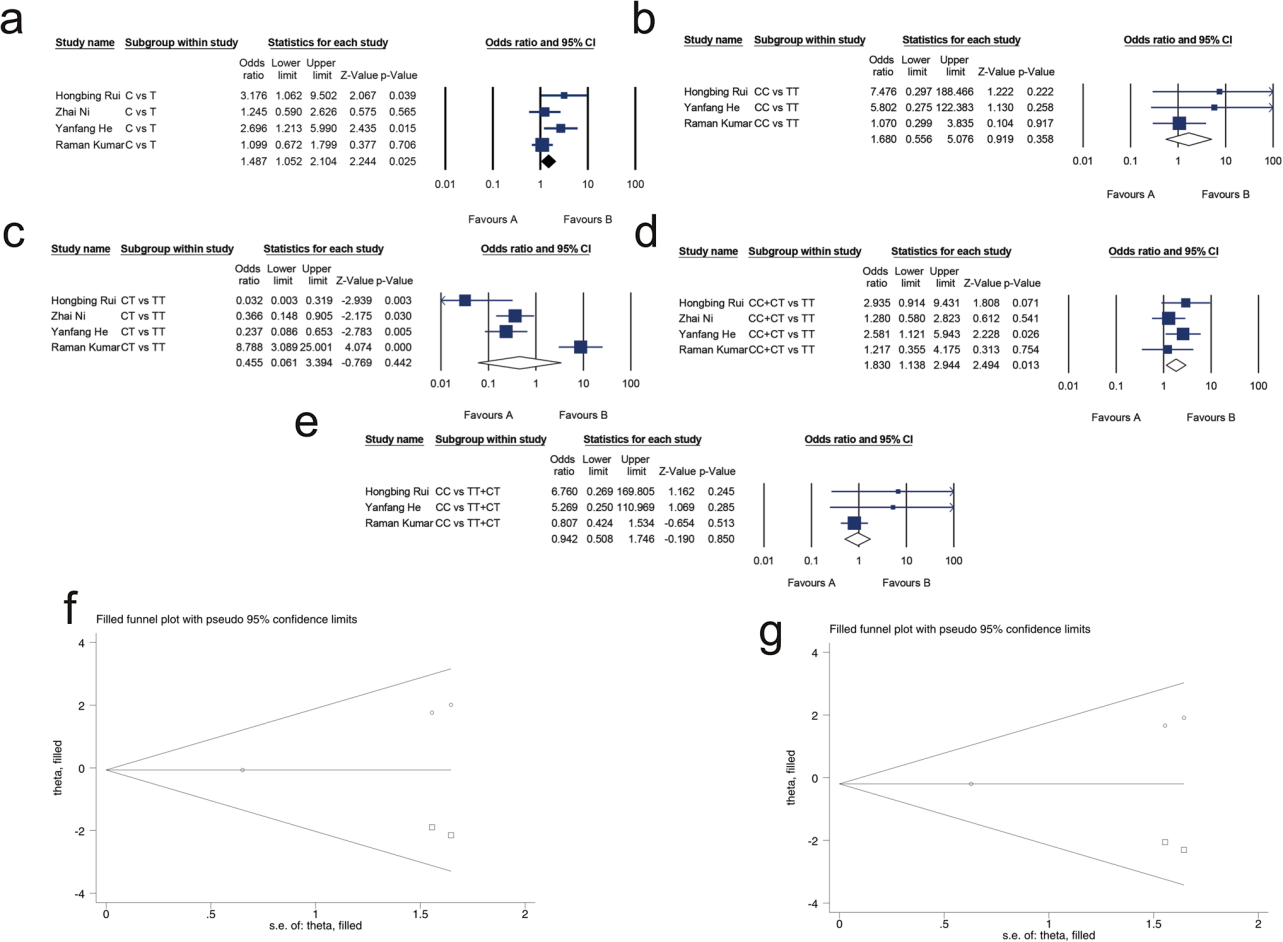
Forest plot of the association between TaqI(rs731236) and multiple myeloma for the allelic model (**a**), homozygote model (**b**), heterozygous model (**c**), dominant model (**d**), and recessive model (**e**). The adjusted funnel plot of the association between TaqI (rs731236) and multiple myeloma after adjustment by the trim-and-fill method for the homozygote model (**f**) and recessive model (**g**). The blank circles represent the original studies, whereas the blanked squares represent the imputed studies

A total of 342 patients and 336 controls were included in the analysis. A summary of the heterogeneity analysis, meta-analysis results, and publication bias assessment is shown in Table 2. No heterogeneity was observed in the allelic model (C vs. T, I^2^ = 46.811%, *P* = 0.130) and dominant model (CC + CT vs. TT, I^2^ = 0.000%, *P* = 0.478), recessive model (CC vs. TT + CT, I^2^ = 30.667%, *P* = 0.236), and homozygote model (CC vs. TT, I^2^ = 0.000%, *P* = 0.380), and high heterogeneity existed in the heterozygous model (CT vs. TT, I^2^ = 91.613%, *P* = 0.000). The results from the meta-analysis showed that TaqI (rs731236) was associated with MM risk in the allelic model (OR 1.487, 95% CI 1.052, 2.104, P = 0.025; Fig. 2a) and dominant model (OR 1.830, 95% CI 1.138, 2.944;* P* = 0.013; Fig. 2d). The results of Begg’s and Egger’s tests showed no publication bias for the allelic model (Egger’s test: *P* = 0.141, Begg’s test: *P* = 0.174) and dominant model (Egger’s test: *P* = 0.205, Begg’s test: *P* = 0.174).

**Table 2:** Meta-analysis of the association between multiple myeloma risk and the TaqI(rs731236)

Genetic model	Test of association			Test of heterogeneity		Test of publication bias	
	OR	95% CI	*P*	I^2^	*P*	Egger’s test (*P*)	Begg’s test (*P*)
C versus T	1.487	1.052, 2.104	0.025	46.811	0.130	0.141	0.174
CC versus TT	1.680	0.556, 5.076	0.358	0.000	0.380	0.012	0.117
CT versus TT	0.455	0.061, 3.394	0.442	91.613	0.000	0.016	0.174
CC + CT versus TT	1.830	1.138, 2.944	0.013	0.000	0.478	0.205	0.174
CC versus TT + CT	0.942	0.508, 1.746	0.850	30.667	0.236	0.012	0.117

According to the primary meta-analysis results, the presence of TaqI (rs731236) was found to be independent of MM in the homozygote model (OR 1.680, 95% CI 0.556, 5.076;* P* = 0.358; Fig. 2b), heterozygous model (OR 0.455, 95% CI 0.061, 3.394;* P* = 0.442; Fig. 2c), and recessive model (OR 0.942, 95% CI 0.508, 1.746; *P* = 0.850; Fig. 2e). However, publication bias was found for the recessive model (Egger’s test: *P* = 0.012, Begg’s test: *P* = 0.117), the homozygote model (Egger’s test:* P* = 0.012, Begg’s test: *P* = 0.117), and the heterozygous model (Egger’s test:* P* = 0.016, Begg’s test: *P* = 0.174). In heterozygous model, heterogeneity decreased (I^2^ = 46.781%, *P* = 0*.*153) following the removal of the data from Raman Kumar [22] from the analysis, and publication bias disappeared (Egger’s test: *P* = 0.059, Begg’s test: *P* = 0.296). Additionally, thetrim-and-fill method was applied to test the possible effect of publication bias, and the results are shown in Table 3. The results tended to be unchanged after adjusting for publication bias using the trim-and-fill method in the homozygote model (OR 0.934, 95% CI 0.232, 3.764; *P* = 0.924; Fig. 2f) and recessive model (OR 0.821, 95% CI 0.312, 2.164; *P* = 0.691; Fig. 2g).

**Table 3:** Trim-and-fill analysis of the eligible studies for the meta-analysis of the association between multiple myeloma risk and TaqI (rs731236)

Genetic model	Test of association			Test of heterogeneity	
	OR	95% CI	*P*	Q	*P*
CC versus TT	0.934	0.232, 3.764	0.924	5.947	0.203
CC versus TT + TC	0.821	0.312, 2.164	0.691	6.142	0.189

#### Association between ApaI (rs7975232) and MM risk

**Fig. 3:**
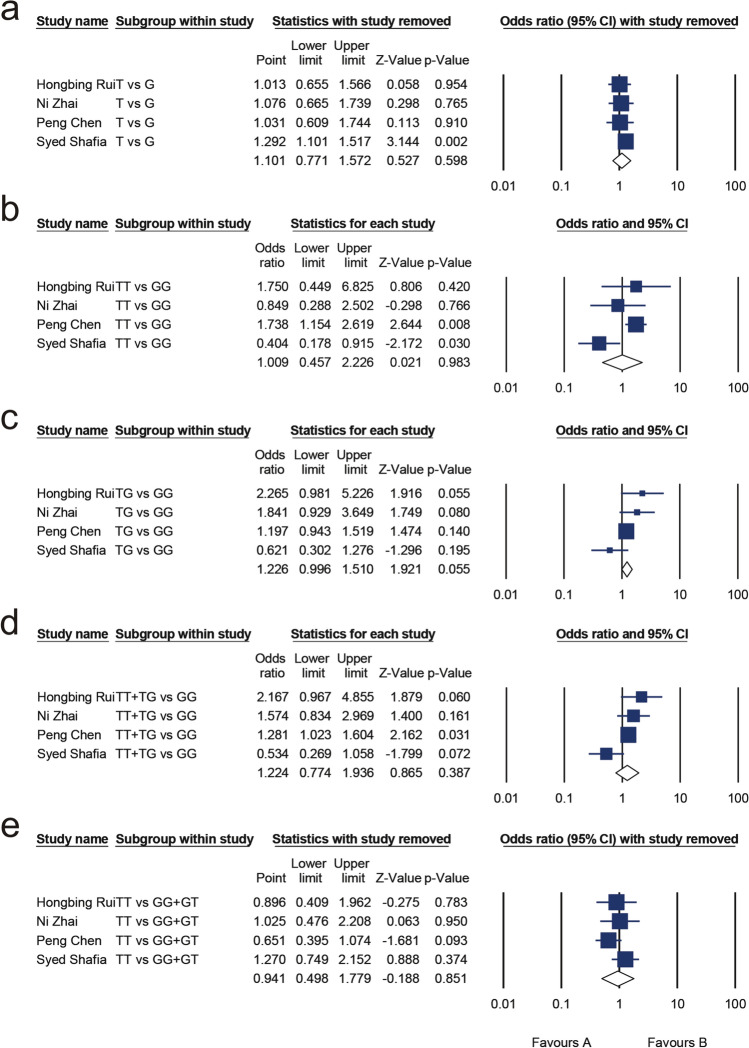
Forest plot for the association between ApaI (rs7975232) and multiple myeloma for the allelic model (**a**), homozygote model (**b**), heterozygous model (**c**), dominant model (**d**), and recessive model (**e**)

A total of 689 cases and 1222 controls were included in the analysis, and publication bias did not exist in any of the genetic model. A summary of the heterogeneity analysis, meta-analysis results, and publication bias assessment is shown in Table 4. Significant heterogeneity existed in the allelic model (T vs. G, I^2^ = 71.414%, *P* = 0.015), homozygote model (TT vs. GG, I^2^ = 71.520%, *P* = 0.015), dominant models (TT + TG vs. GG, I^2^ = 63.766%, *P* = 0.041), heterozygous model (TG vs. GG, I^2^ = 56.475%, P = 0.075), and recessive model (TT vs. GG + GT, I^2^ = 65.229%, *P* = 0.043). The primary meta-analysis results showed that ApaI is not associated with MM risk in the allelic model (OR 1.101, 95% CI 0.771, 1.572;* P* = 0.598; Fig. 3a), homozygote model (OR 1.009, 95% CI 0.457, 2.226; *P* = 0.983; Fig. 3b), heterozygous model (OR 1.226, 95% CI 0.996, 1.510; P = 0.055; Fig. 3c), dominant model (OR 1.224, 95% CI 0.774, 1.936; *P* = 0.387; Fig. 3d), recessive model (OR 0.941, 95% CI 0.498, 1.779; *P* = 0.851; Fig. 3e). We further searched for sources of heterogeneity by conducting a sensitivity analysis, which was carried out by removing studies one by one. Heterogeneity was reduced after the elimination of the data from Syed Shafia [24], and statistical significance was obtained for the allelic model (OR 1.292, 95% CI 1.101, 1.517; *P* = 0.002), dominant model (OR 1.353, 95% CI 1.103, 1.662; *P* = 0.004), heterozygous model (OR 1.305, 95% CI 1.050, 1.622; *P* = 0.016), and homozygote model (OR 1.600, 95% CI 1.106, 2.314; *P* = 0.013). The heterogeneity in the recessive model decreased to 26.291% after excluding the study of Syed Shafia, and the results of the meta-analysis remained statistically insignificant (OR 1.411, 95% CI 0.988, 2.017; *P* = 0.058). The results are shown in Table 5.

**Table 4:** Meta-analysis of the association between multiple myeloma risk and the ApaI (rs7975232)

Genetic model	Test of association			Test of heterogeneity		Test of publication bias	
	OR	95% CI	*P*	I^2^	*P*	Egger’s test (*P*)	Begg’s test (*P*)
T versus G	1.101	0.771, 1.572	0.598	71.414	0.015	0.691	1.000
TT versus GG	1.009	0.457, 2.226	0.983	71.520	0.015	0.483	1.000
TG versus GG	1.226	0.996, 1.510	0.055	56.475	0.075	0.797	0.496
TT + TG versus GG	1.224	0.774, 1.936	0.387	63.766	0.041	0.921	0.496
TT versus GG + TG	0.941	0.498, 1.779	0.851	65.229	0.043	0.406	1.000

**Table 5:** Meta-analysis of the association between multiple myeloma risk and the ApaI (rs7975232) after the removal of the study by Syed Shafia

Genetic model	Test of association			Test of heterogeneity		Test of publication bias	
	OR	95% CI	*P*	I^2^	*P*	Egger’s test (*P*)	Begg’s test (*P*)
T versus G	1.292	1.101, 1.517	0.002	0.000	0.791	0.757	0.601
TT versus GG	1.600	1.106, 2.314	0.013	0.000	0.473	0.587	0.601
TG versus GG	1.305	1.050, 1.622	0.016	36.516	0.207	0.290	0.518
TT + TG versus GG	1.353	1.103, 1.662	0.004	0.000	0.416	0.783	0.117
TT versus GG + TG	1.411	0.988, 2.017	0.058	26.291	0.258	0.363	0.601

#### Association between BsmI (rs1544410) and MM risk

**Fig. 4:**
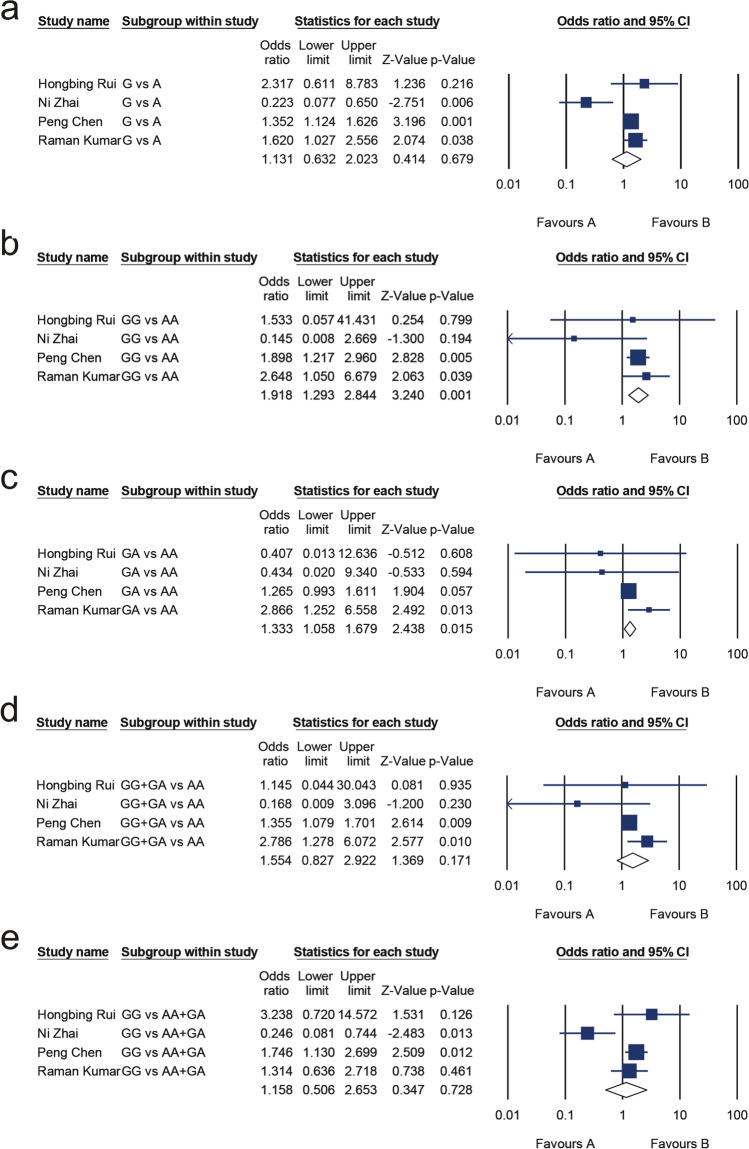
Forest plots of the association of BsmI (rs1544410) and multiple myeloma for the allelic model (**a**), homozygote model (**b**), heterozygous model (**c**), dominant model (**d**), and recessive model (**e**)

Six hundred eighty-nine patients with MM and 1147 healthy control subjects were included. A summary of the heterogeneity results, meta-analysis results, and publication bias assessment is shown in Table 6. The results of Begg’s and Egger’s tests showed no publication bias. Heterogeneity tests revealed significant heterogeneity in the allelic model (G vs. A, I^2^ = 75.272%, *P* = 0.007) and recessive model (GG vs. AA + GA, I^2^ = 74.332%, *P* = 0.009). Primary meta-analysis results showed that the allelic model (OR 1.131, 95% CI 0.632, 2.023; *P* = 0.679; Fig. 4a) and recessive model (OR 1.158, 95% CI 0.506, 2.653; *P* = 0.728; Fig. 4e) were independent of MM risk. But heterogeneity was high, so sensitivity analysis was carried out by removing trials one by one. Removal of the data from Ni Zhai [21] reduced the heterogeneity of allelic model and dominant model to zero, and the results of meta-analysis became statistically significant (allelic model: OR 1.398, 95% CI 1.180, 1.657, *P* = 0.000; recessive model: OR 1.686, 95% CI 1.174, 2.423; *P* = 0.005), as shown in Table 7.

**Table 6:** Meta-analysis of the association between multiple myeloma risk and BsmI (rs1544410)

Genetic model	Test of association			Test of heterogeneity		Test of publication bias	
	OR	95% CI	*P*	I^2^	*P*	Egger’s test (*P*)	Begg’s test (*P*)
G versus A	1.131	0.632, 2.023	0.679	75.272	0.007	0.688	1.000
GG versus AA	1.918	1.293, 2.844	0.001	14.454	0.320	0.470	0.496
GA versus AA	1.333	1.058, 1.679	0.015	32.359	0.218	0.749	1.000
GG + GA versus AA	1.554	0.827, 2.922	0.171	41.331	0.164	0.605	0.496
GG versus AA + GA	1.158	0.506, 2.653	0.728	74.332	0.009	0.928	1.000

**Table 7:** Meta-analysis of the association between multiple myeloma risk and BsmI (rs1544410) polymorphism after the removal of the study by Ni Zhai

Genetic model	Test of association			Test of heterogeneity		Test of publication bias	
	OR	95% CI	*P*	I^2^	*P*	Egger’s test (*P*)	Begg’s test (*P*)
G versus A	1.398	1.180, 1.657	0.000	0.000	0.582	0.113	0.117
GG versus AA + GA	1.686	1.174, 2.423	0.005	0.000	0.549	0.723	0.601

There was no obvious heterogeneity in the homozygote model (GG vs. AA, I^2=^14.454%, *P* = 0.320), heterozygous model (GA vs. AA, I^2^ = 32.359%, *P* = 0.218), and dominant model (GG + GA vs. AA, I^2^ = 41.331%, *P* = 0.164), so the fixed-effect model was selected. The meta-analysis indicated a highly significant association between BsmI and the risk of MM for the homozygote model (OR 1.918, 95% CI 1.293, 2.844; *P* = 0.001; Fig. 4b) and heterozygous model (OR 1.333, 95% CI 1.058, 1.679; *P* = 0.015; Fig. 4c), but there was no association between BsmI and MM in dominant model (OR 1.554, 95% CI 0.827, 2.922; *P* = 0.171; Fig. 4d).

#### Association between FokI(rs2228570) and MM risk

**Fig. 5:**
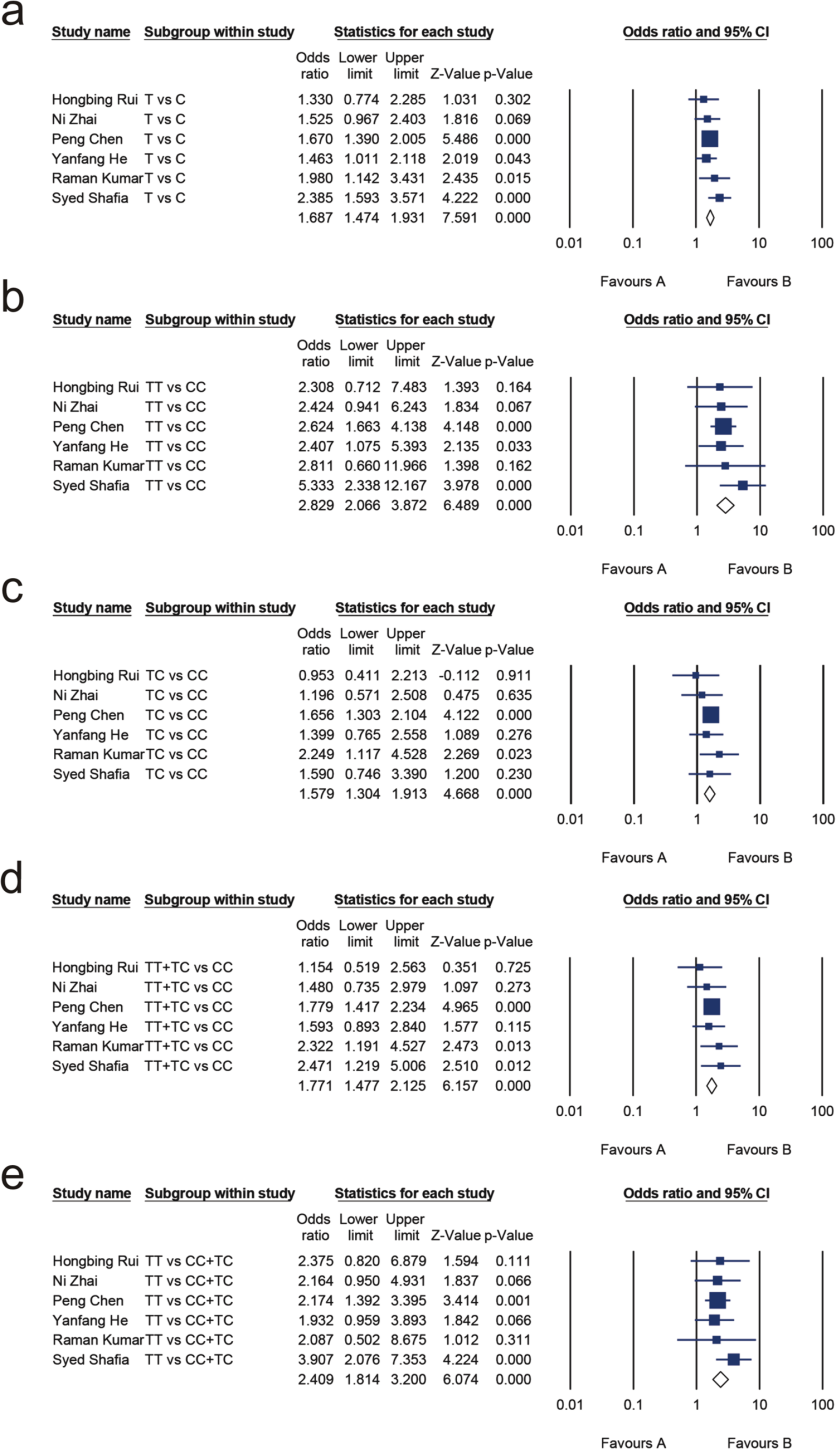
Forest plots for the association between FokI (rs2228570) and multiple myeloma risk for the allelic model (**a**), homozygote model (**b**), heterozygous model (**c**), dominant model (**d**), and recessive model (**e**)

There were 877 patients with MM and 1414 healthy controls involved in the study. A summary of the heterogeneity analysis, meta-analysis results, and publication bias assessment is shown in Table 8. No substantial heterogeneity existed in the allelic model (T vs. C, I^2^ = 0.000%, *P* = 0.458), dominant models (TT + CT vs. CC, I^2^ = 0.000%, *P* = 0.703), recessive model (TT vs. CC + CT, I^2^ = 0.000%, *P* = 0.710), homozygote model (TT vs. CC, I^2^ = 0.000%, *P* = 0.739), and heterozygous model (CT vs. CC, I^2^ = 0.000%, *P* = 0.668), and the test for publication bias was not significant. Meta-analysis found that FokI is associated with MM risk in the allelic model (OR 1.687, 95% CI 1.474, 1.931; *P* = 0.000; Fig. 5a), homozygote model (OR 2.829, 95% CI 2.066, 3.872; *P* = 0.000; Fig. 5b), heterozygous model (OR 1.579, 95% CI 1.304, 1.913; *P* = 0.000; Fig. 5c), dominant model (OR 1.771, 95% CI 1.477, 2.125; *P* = 0.000; Fig. 5d), and recessive model (OR 2.409, 95% CI 1.814, 3.200; *P* = 0.000; Fig. 5e).

**Table 8:** Meta-analysis of the association between multiple myeloma risk and FokI (rs2228570)

Genetic model	Test of association			Test of heterogeneity		Test of publication bias	
	OR	95% CI	*P*	I^2^	*P*	Egger’s test (*P*)	Begg’s test (*P*)
T versus C	1.687	1.474, 1.931	0.000	0.000	0.458	0.940	0.850
TT versus CC	2.829	2.066, 3.872	0.000	0.000	0.739	0.876	0.188
TC versus CC	1.579	1.304, 1.913	0.000	0.000	0.668	0.819	0.850
TT + TC versus CC	1.771	1.477, 2.125	0.000	0.000	0.703	0.947	0.573
TT versus CC + TC	2.409	1.814, 3.200	0.000	0.000	0.710	0.882	0.573

## Discussion

Low level of vitamin D is related to increased risk of several cancers [28]. Vitamin D regulates the process of oncogenesis through immunomodulation, antioxidant defense, and DNA damage repair, which affects cancel cell proliferation and apoptosis [29]. Previous studies have investigated the relationship between VDR gene polymorphisms and MM [19–24]. However, the results of different studies are controversial. We therefore performed this meta-analysis to comprehensively evaluate these inconclusive findings.

TaqI (rs731236) is located at codon 352 in exon 9 of VDR gene, which creates a TaqI restriction site and related to transcriptional activity, mRNA stability, and the serum level of 1,25(OH)_2_D_3_ [30, 31]. Previous studies have reported that MM patients have a significantly greater frequency of the C allele at the TaqI than healthy controls [19, 23], but other studies have reached inconsistent conclusions [21, 22]. We included 342 patients and 336 controls from four studies. The results from this meta-analysis showed that TaqI is associated with MM in the dominant model and heterozygous model but is independent of MM risk in the allelic model, homozygote model, and recessive model.

ApaI (rs7975232) is positioned in intron 8 near the 3′ end of VDR gene and has the potential to affect alternative splicing of the VDR mRNA [32]. Four studies did not support the association between ApaI and MM risk [20, 21, 23, 24], but one study reached the opposite conclusion [22]. In this meta-analysis, 689 patients and 1222 controls were included. There was heterogeneity in the primary analysis, and the heterogeneity disappeared after the elimination of the study from Syed Shafia [24]. Syed Shafia’s study including individuals of Kashmiri ethnicity, while the subjects of the remaining four study subjects were of the Chinese Han ethnicity, which suggests that ethnicity might be the source of the heterogeneity. The meta-analysis results showed that ApaI is correlated with MM risk in the allelic model, dominant model, and homozygote model.

Previous studies reported that BsmI (rs1544410) is located at the 3’ end of the noncoding region of VDR gene, and enhances the stability and transcriptional activity of VDR gene [30]. Previous studies reported that BsmI is significantly associated with MM risk [21–23], but one study reported that there is no association [20]. This meta-analysis included 689 cases and 1147 healthy controls. Removing the data from Ni Zhai’s study [21] reduced the heterogeneity in the allelic model and dominant model, and the meta-analysis indicated a highly significant association between BsmI and the risk of MM in the allelic model, recessive model, homozygote model and heterozygous model.

FokI (rs2228570) is located in exon 2 of VDR gene. The association between FokI polymorphism and increased MM risk was described in four studies [19, 20, 22, 24], whereas opposite results were reported in the other two studies [21, 23]. In this meta-analysis, we pooled data of 877 MM patients and 1414 healthy controls from six studies. The meta-analysis revealed that FokI is associated with MM risk in the allelic model, dominant model, recessive model, homozygote model, and heterozygous model. Collectively, individuals with the T genotype had a significantly greater risk of MM than those with the C genotype. Previous studies have reported that the C to T conversion causes shortening of the generated proteins by three amino acids and the loss of the FokI-recognition site, causing the VDR to be less effective as a transcriptional activator and resulting in a lower vitamin D status, thus, decreasing the anti-cancer effects of vitamin D [33].

In this study, a meta-analysis to evaluate the polymorphisms of VDR gene (FokI, BsmI and ApaI) and MM risk was performed for the first time, and TaqI, FokI, ApaI, and BsmI polymorphisms were found to be associated with MM risk. However, SNPs have geographical and ethnic differences; the results of this meta-analysis may be difficult to extrapolate to non-Asian populations because the included studies examining the association between VDR polymorphisms and MM were limited to Asian participants. The association between VDR gene polymorphisms and MM has not been widely studied, resulting in the relatively small number of included studies. Further high-quality studies with multiple ethnicities and larger sample sizes are needed. Although a meta-analysis of VDR gene polymorphisms and MM risk provides valuable information, we must be cautious in how interpret these associations and continue to conduct in-depth research to reveal the potential underlying biological mechanism involved.

## Supplementary Information

Below is the link to the electronic supplementary material.

Supplementary file 1 (DOCX 21 kb)

Supplementary file 2 (DOCX 16 kb)

Supplementary file 3 (DOCX 19 kb)

## Data Availability

Supporting data are available from the corresponding author on reasonable request.
