# Supplementary material for: Vitamin D receptor gene polymorphisms and multiple myeloma: a meta-analysis
Source: Clin Exp Med. 2024 Jun 4;24(1):118. doi: 10.1007/s10238-024-01382-4 (PMC11150203; doi:10.1007/s10238-024-01382-4)
Supplement: Supplementary file 2 — (DOCX 16 kb) [file 10238_2024_1382_MOESM2_ESM.docx]

**Supplementary file 2.** Search strategies for English and Chinese databases.

| Database | Search strategy |
| --- | --- |
| PubMed | (multiple myeloma[MeSH Terms]) AND ((vitamin D receptor[Title/Abstract]) OR (VDR[Title/Abstract])) AND (polymorphism) |
| Web of Science | TI=(multiple myeloma) AND (TI=(vitamin D receptor) OR AB=(vitamin D receptor) AND TI=(VDR) OR AB=(VDR)) AND ALL=(polymorphism) |
| Medline | TI multiple myeloma AND (TI vitamin d receptor OR AB vitamin d receptor AND TI vdr OR AB vdr) AND TX polymorphism |
| Embase | ('vitamin d receptor':ab,ti OR 'vdr':ab,ti) AND polymorphism AND 'multiple myeloma':ti |
| CNKI | TI=多发性骨髓瘤 AND (AB=维生素D受体 OR TI = 维生素D受体) AND (FT=单核苷酸多态性 OR FT=基因多态性) |
| VIP | M=多发性骨髓瘤 AND (R=维生素D受体 OR M= 维生素D受体) AND (U=单核苷酸多态性 OR U=基因多态性) |
| WANFANG | 多发性骨髓瘤 and (题名或关键词:维生素D受体 or 摘要:维生素D受体) and (全部:单核苷酸多态性 or 全部:基因多态性) |
| CNKI: Chinese National Knowledge Infrastructure; VIP: Chinese Scientific Journal Database; WANFANG: Wanfang Database. | |
