# Supplementary material for: Vitamin D receptor gene polymorphisms and multiple myeloma: a meta-analysis
Source: Clin Exp Med. 2024 Jun 4;24(1):118. doi: 10.1007/s10238-024-01382-4 (PMC11150203; doi:10.1007/s10238-024-01382-4)
Supplement: Supplementary file 3 — (DOCX 19 kb) [file 10238_2024_1382_MOESM3_ESM.docx]

**Supplementary file 3.** Genotypic characterization of the included studies.

| First Author, Publication time | Case | | | | | Control | | | | | HWE of control (*P*) |
| --- | --- | --- | --- | --- | --- | --- | --- | --- | --- | --- | --- |
| TaqI (rs731236) | TT | CC | CT | T | C | TT | CC | CT | T | C |  |
| Hongbing Rui, 2018 | 31 | 1 | 6 | 68 | 8 | 78 | 0 | 6 | 162 | 6 | 0.734 |
| Ni Zhai, 2018 | 87 | 0 | 25 | 199 | 25 | 49 | 0 | 11 | 109 | 11 | 0.434 |
| Yanfang He, 2017 | 93 | 2 | 18 | 204 | 22 | 108 | 0 | 9 | 225 | 9 | 0.665 |
| Raman Kumar, 2020 | 33 | 5 | 37 | 103 | 47 | 37 | 6 | 32 | 106 | 44 | 0.800 |
| ApaI (rs7975232) | TT | GG | GT | T | G | TT | GG | TG | T | G |  |
| Hongbing Rui, 2018 | 4 | 12 | 22 | 30 | 46 | 8 | 42 | 34 | 50 | 11 | 0.770 |
| Ni Zhai, 2018 | 9 | 50 | 53 | 71 | 153 | 7 | 33 | 19 | 33 | 85 | 0.123 |
| Peng Chen, 2017 | 47 | 238 | 175 | 269 | 651 | 61 | 537 | 330 | 452 | 1404 | 0.288 |
| Syed Shafia, 2013 | 17 | 19 | 39 | 73 | 77 | 51 | 23 | 76 | 178 | 122 | 0.540 |
| BsmI (rs1544410) | AA | GG | GA | G | A | AA | GG | GA | G | A |  |
| Hongbing Rui, 2018 | 1 | 34 | 5 | 73 | 7 | 0 | 7 | 4 | 1 | 4 | 0.461 |
| Ni Zhai, 2018 | 5 | 83 | 21 | 187 | 31 | 0 | 52 | 4 | 108 | 4 | 0.781 |
| Peng Chen, 2017 | 259 | 40 | 161 | 242 | 679 | 590 | 48 | 290 | 386 | 1470 | 0.117 |
| Raman Kumar, 2020 | 12 | 22 | 41 | 85 | 65 | 26 | 18 | 31 | 67 | 83 | 0.155 |
| FokI (rs2228570) | TT | CC | CT | T | C | TT | CC | CT | T | C |  |
| Hongbing Rui, 2018 | 8 | 13 | 19 | 35 | 45 | 8 | 30 | 46 | 62 | 106 | 0.106 |
| Ni Zhai, 2018 | 31 | 27 | 51 | 113 | 105 | 9 | 19 | 30 | 48 | 68 | 0.614 |
| Peng Chen, 2017 | 42 | 237 | 181 | 265 | 655 | 41 | 607 | 280 | 362 | 1494 | 0.233 |
| Yanfang He, 2017 | 25 | 27 | 61 | 111 | 115 | 15 | 39 | 63 | 93 | 141 | 0.179 |
| Raman Kumar, 2020 | 6 | 37 | 32 | 44 | 106 | 3 | 52 | 20 | 26 | 124 | 0.547 |
| Syed Shafia, 2013 | 32 | 12 | 31 | 95 | 55 | 24 | 48 | 78 | 126 | 174 | 0.409 |
| HWE: Hardy–Weinberg equilibrium. | | | | | | | | | | | |
